# Expressed sequence tags (ESTs) from immune tissues of turbot (*Scophthalmus maximus*) challenged with pathogens

**DOI:** 10.1186/1746-6148-4-37

**Published:** 2008-09-25

**Authors:** Belén G Pardo, Carlos Fernández, Adrián Millán, Carmen Bouza, Araceli Vázquez-López, Manuel Vera, José A Alvarez-Dios, Manuel Calaza, Antonio Gómez-Tato, María Vázquez, Santiago Cabaleiro, Beatriz Magariños, Manuel L Lemos, José M Leiro, Paulino Martínez

**Affiliations:** 1 Departamento de Genética. Facultad de Veterinaria, Universidad de Santiago de Compostela, Campus de Lugo, 27002 Lugo, Spain; 2 Departamento de Matemática Aplicada, Facultad de Matemáticas, Universidad de Santiago de Compostela, 15782 Santiago de Compostela, Spain; 3 Departamento de Geometría y Topología, Facultad de Matemáticas, Universidad de Santiago de Compostela, 15782 Santiago de Compostela, Spain; 4 Cluster de la Acuicultura de Galicia (CETGA), Punta de Couso s/n, 15965, Aguiño (Ribeira), A Coruña, Spain; 5 Departamento de Microbiología y Parasitología. Universidad de Santiago de Compostela, 15782 Santiago de Compostela, Spain

## Abstract

**Background:**

The turbot (*Scophthalmus maximus*; Scophthalmidae; Pleuronectiformes) is a flatfish species of great relevance for marine aquaculture in Europe. In contrast to other cultured flatfish, very few genomic resources are available in this species. *Aeromonas salmonicida *and *Philasterides dicentrarchi *are two pathogens that affect turbot culture causing serious economic losses to the turbot industry. Little is known about the molecular mechanisms for disease resistance and host-pathogen interactions in this species. In this work, thousands of ESTs for functional genomic studies and potential markers linked to ESTs for mapping (microsatellites and single nucleotide polymorphisms (SNPs)) are provided. This information enabled us to obtain a preliminary view of regulated genes in response to these pathogens and it constitutes the basis for subsequent and more accurate microarray analysis.

**Results:**

A total of 12584 cDNAs partially sequenced from three different cDNA libraries of turbot (*Scophthalmus maximus*) infected with *Aeromonas salmonicida*, *Philasterides dicentrarchi *and from healthy fish were analyzed. Three immune-relevant tissues (liver, spleen and head kidney) were sampled at several time points in the infection process for library construction. The sequences were processed into 9256 high-quality sequences, which constituted the source for the turbot EST database. Clustering and assembly of these sequences, revealed 3482 different putative transcripts, 1073 contigs and 2409 singletons. BLAST searches with public databases detected significant similarity (e-value ≤ 1e-5) in 1766 (50.7%) sequences and 816 of them (23.4%) could be functionally annotated. Two hundred three of these genes (24.9%), encoding for defence/immune-related proteins, were mostly identified for the first time in turbot. Some ESTs showed significant differences in the number of transcripts when comparing the three libraries, suggesting regulation in response to these pathogens. A total of 191 microsatellites, with 104 having sufficient flanking sequences for primer design, and 1158 putative SNPs were identified from these EST resources in turbot.

**Conclusion:**

A collection of 9256 high-quality ESTs was generated representing 3482 unique turbot sequences. A large proportion of defence/immune-related genes were identified, many of them regulated in response to specific pathogens. Putative microsatellites and SNPs were identified. These genome resources constitute the basis to develop a microarray for functional genomics studies and marker validation for genetic linkage and QTL analysis in turbot.

## Background

The turbot (*Scophthalmus maximus*; Scophthalmidae; Pleuronectiformes) is a commercially valuable flatfish that has been intensively cultured for the last decade. Its production has steadily increased to 7120 tonnes in 2006 (80% from Spain; FEAP, 2006) and represents one of the most promising aquaculture species in Europe. However, disease outbreaks in turbot have occurred frequently and losses due to infections constitute a serious problem for its culture [1,2]. The use of antibiotics, vaccines and fish health management practices has partially solved the problem, but the achievement of large-scale production in the highly competitive world market requires enhancing resistance of cultured fish to diseases. Information on the immune response of turbot is still limited, and little is known about host-pathogen interactions in fish species. The screening and identification of immune-relevant genes is essential to analyze the genetic basis for infection, immunity and resistance to pathogens of economic relevance in aquaculture. Expressed sequence tag (EST) analysis is a powerful approach to provide a rapid and efficient method to go from expressed sequences to genes. ESTs are essential for studies of gene function [3,4], but are also useful to identify polymorphic gene markers, such as microsatellites and single nucleotide polymorphisms (SNPs) [5-8]. These markers are the basis for genetic and physical mapping, and for comparative genome analysis [9-11]. From a practical perspective, maps can be applied for assisted selection programmes (MAS) and eventually for identification of genes related with quantitative traits (QTL) [12,13]. In addition, ESTs constitute the basic resources to develop microarrays for functional genomics studies [14].

EST sequence resources are rapidly growing in molecular databases. However, the number of ESTs in fish is generally scarce, excluding some model species and Atlantic salmon among cultured fish [15-19]. The scarcity of EST resources in cultured fish limits the use of modern functional genomic approaches for selective breeding purposes [20]. Among flatfish, aquaculture production has been successfully achieved in turbot, Japanese flounder and Atlantic halibut. Compared to the very large efforts for the development of EST resources in Japanese flounder (8856 ESTs) and Atlantic halibut (17659 ESTs) [21-28], EST resources in turbot are scarce (3171 ESTs). Less than 800 sequences have been deposited to date in the NCBI nucleotide database, most of them from anonymous microsatellite searching [29].

With the aim of increasing the genomic resources in turbot and identifying relevant genes for immunity, three cDNA libraries were constructed from mRNA isolated from immunity-related tissues of turbot (liver, spleen and head kidney) at different times after infection with *Aeromonas salmonicida *and the scuticociliate parasite *Philasterides dicentrarchi*. These pathogens are responsible for important disease outbreaks in turbot, as well as in other culture fish species [30-33]. Our main goal was to obtain the most accurate information possible to address functional genomic studies on disease resistance. However, the use of non-normalized cDNA libraries made it feasible to get a preliminary picture of the turbot genetic response to pathogens through analyzing transcript distribution among infected *vs*. control libraries. A total of 12584 ESTs were sequenced and compared to GenBank database and a large array of defence or immune-related genes was identified. Also, this large scale EST study increased the number of putative markers for mapping. A total of 191 microsatellites, of which 104 exhibited sufficient flanking sequences for primer design, and 2197 good quality SNPs were identified for the first time in turbot. The cDNA sequences generated will serve as a basis for microarray construction. This first EST study in turbot will provide the support for further research into the genetics, genomics and even proteomics of this important aquaculture species.

## Results and Discussion

### cDNA libraries and ESTs

EST analysis is an efficient and fast method for gene discovery [15,17]. In Pleuronectiformes, this approach has been recently applied in Japanese flounder (*Paralichthys olivaceus*; [21,22,24,25]), winter flounder (*Pseudopleuronectes americanus*; [34]), flounder (*Platichthys flesus*; [35,36]) and Atlantic halibut (*Hippoglossus hippoglossus*; [26-28]. However, this order comprises around 600 species, many of them of great economic value both for fisheries and farming. In this study, we have addressed the construction of an EST database for the identification of genes related with immunity and defence in turbot.

The three cDNA libraries constructed held at least 2.5 × 10^6 ^primary recombinant clones. The directional cloning approach used for construction of cDNA libraries ensured that cDNA inserts appeared mostly in the same orientation within the vector. Clones were sequenced from the 3' end with the vector primer T7 to obtain large gene-specific genomic regions for future oligo-microarray design. The libraries used were non-normalized and, as usually observed [16], substantial redundancy was obtained (around 74%). This approach allowed an analysis of the turbot response to specific pathogens by comparing the amount of transcripts across all genes or groups of genes classified in functional categories. A total of 12584 ESTs were sequenced. After trimming and vector removal 9256 high quality ESTs were obtained (Table 1), showing an average length of 409 bp. Their sequences are available in the dbEST NCBI database under numbers FE943103-FE952358.

**Table 1:** Summary statistics of ESTs from turbot libraries

	Number	%
Good-quality ESTs	9256	
Redundant sequences	6847	74.0
Unique sequences	3482	36.0
Contigs	1073	30.8
Singletons	2409	69.2
Unique sequences with no BLAST hits	1716	49.3
Unique sequences with BLAST hits	1766	50.7
BLASTN	1091	61.8
BLASTX	675	38.2
Unique sequences with functional annotation	816	23.4
Contigs	489	59.9
Singletons	327	40.1

**Figure 1:**
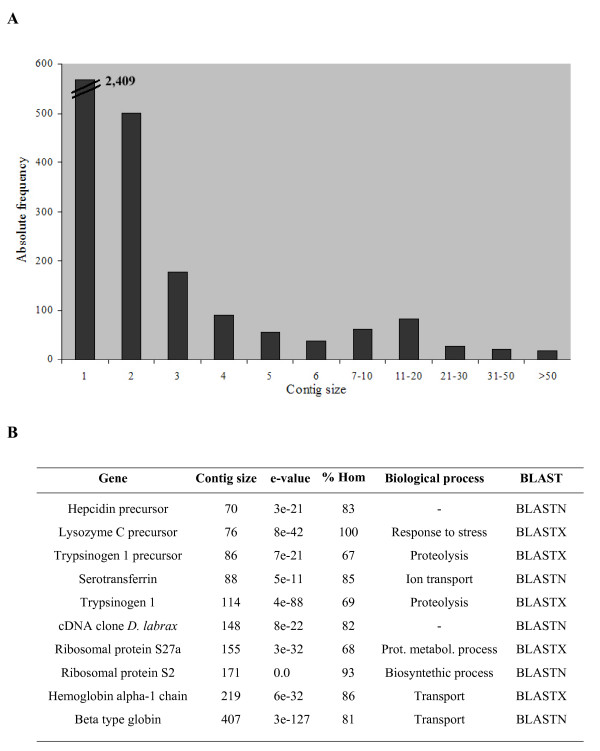
**Sequence prevalence distribution of the identified contigs from turbot libraries**. (A) Absolute frequency histogram showing contig size (number of sequences) distribution. (B) Functional and BLAST hits confidence characteristics of the ten largest contigs. Only biological function according to GO terms has been included.

EST projects generate a large number of redundant sequences due to the random selection of cDNAs from tissue libraries, especially when libraries are non-normalized and a high number of clones are sequenced. Clustering redundant sequences is a critical step to identify genes. The program CAP3  was used to cluster EST sequences using the default parameters. As shown in Table 1, clustering yielded 3482 unique turbot sequences: 2409 singletons (69.2%) and 1073 contigs (30.8%) comprising 6847 ESTs (6.4 sequences/contig) and an average length of 527 bp. Figure 1A shows the histogram distribution of contig sizes. Although most contigs showed two (46.7%) or three-to-five (30.3%) sequences, a small number of highly expressed genes were also detected. As shown in Figure 1B, the beta-globin contig (407 ESTs) and five others contained more than 100 ESTs, which represents a high proportion (1212/9256 = 13.1%) over the total redundancy (74.0%). Most of these ESTs shared homology with genes involved in transport, protein metabolism and response to stress, all of which related to defence. At the other extreme, 77.0% of the 1073 contigs were ≤ 5 times redundant, indicating that most of these unique sequences represent rare mRNAs and that these libraries provide a rich source of sequence information.

### Gene annotation

ESTs were identified by BLAST searches against nucleotide database at GenBank. Due to the low representation of fish genes, a protein-based homology strategy was also used in the international database searches. Protein sequences have been demonstrated to be more suitable to detect homology over long periods of evolutionary time [37]. Our EST database pprovided a graphical view of all contigs, their PROSITE/PRINTS protein patterns and a search interface by keywords, microsatellite, gene, and UniGene/GO/KEGG information. Tools to search for sequences and markers based on annotations, to perform local BLAST searches, and to select sequences for a prospective microarray were also included. These tools used RepeatMasker  for masking low-complexity sequences and OligoArray 2.1  to predict secondary structures and potential cross-hybridization.

As shown in Table 1, 1716 (49.3%) unique sequences displayed no significant similarity to known sequences or ESTs in the public databases, whereas the remaining 1766 (50.7%) showed significant matches with e-values ≤ 1e-5. Among these, BLAST database searches allowed assignment of putative function to 816 sequences (23.4%). In spite of their lower frequency among unique sequences, contigs were annotated more frequently (59.9%) than singletons (40.1%). As in other ESTs fish studies, the lower percentage of annotated singletons suggests that these are either novel fish-specific or rapidly evolving genes [38]. Also, it is possible that bioinformatic errors could have a greater impact on singletons, since they are unique sequences whose information cannot be contrasted with other sequences in the database. The lower annotation success regarding similar genomic projects [28,39] was probably related to the read length (around 500 bp) and specially the direction (from the 3'end) of sequencing. The 3' untranslated region (UTR) is approximately double the length of the 5'UTR according to GenBank fish entries. So, what is gained in specificity for microarray oligo-design is lost for gene annotation.

All unique sequences were annotated based on similarity using BLASTX or BLASTN [40] in the public databases GenBank NR and Unigene. The multiple annotations provided greater assurance about gene description and frequency of annotation than in a single database. The use of consensus sequences allowed sequences without significant similarity regions with a known protein (e.g., 5' or 3' noncoding regions) to be annotated if they were members of an annotated contig. All hits with e-value ≤ 1e-5 and their associated alignments were stored in the database and tracked with any associated functional annotation. We also ran AutoFACT [41] on all sequences in the database. It is interesting to note that while AutoFACT was able to come up with more function-specific information than our online tool (provided external database hits were found), it was not able to annotate as many sequences as our online custom tool.

**Figure 2:**
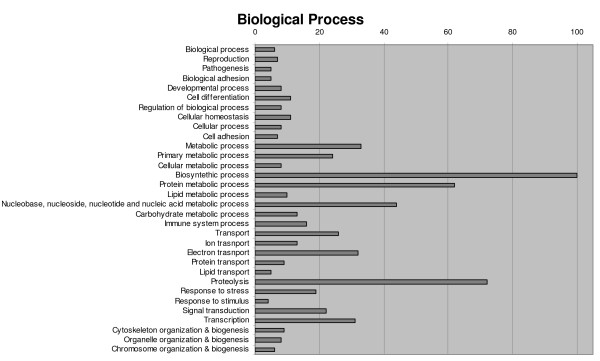
Classification of turbot unique sequences in biological processes categories following Gene Ontology (GO).

**Figure 3:**
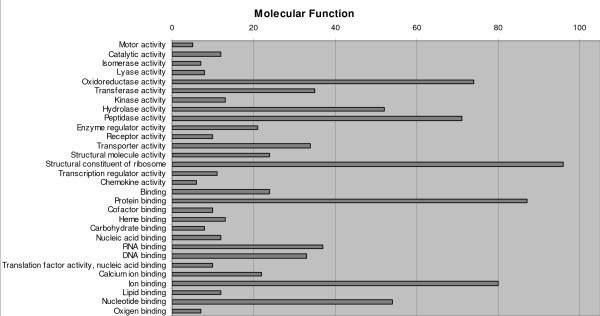
Classification of turbot unique sequences in molecular function categories following Gene Ontology (GO).

**Figure 4:**
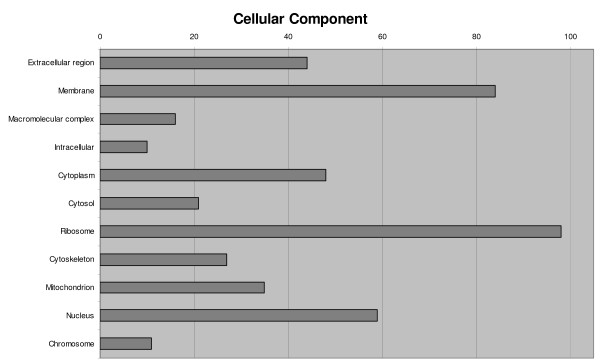
Classification of turbot unique sequences in cellular component categories following Gene Ontology (GO).

Annotated ESTs (816) were classified into functional categories according to GO terms [42] (77.0%), 367 among contigs (75.1%) and 261 among singletons (79.8%). A single sequence very often showed several GO terms in the same ontology, so we try to group them using a single more general category. Overly specific categories were also collapsed into more general terms. Any terms related to defence/immunity were always retained given their interest in this study. Biological process, molecular function and cellular component categories are shown in Figures 2, 3 and 4, respectively. According to these criteria, biological processes associated with proteins (synthesis, metabolism and proteolysis) were the largest annotated categories, though a significant group related to transport and response to stress did appear. In accordance with this, a large proportion of sequences were classified into structural constituent of ribosome and protein-related functions (binding, peptidase activity) regarding molecular function. A remarkably high proportion of annotated sequences were categorized as oxidoreductase activity. Finally, most gene activity was located into the ribosome, followed by the membrane, nucleus and the extracellular and cytoplasm constituents of the cell.

### Immune genes

Systematic classification of annotated sequences using available bioinformatics tools (GO, KEGG) provides a useful information to analyze the functional profile of annotated genes. In our study, we were especially interested in identifying immune genes or in a broader sense those genes regulated in response to specific pathogens of turbot. So, we analyzed the relationship of our annotated genes with defence or immunity using scientific literature information available on vertebrates. According to this analysis, 203 genes out of 816 annotated (24.9%) appeared related to defence or immunity in our work (Table 2). This observation is in agreement with the important role of liver, spleen and head kidney in the immune response of fish.

**Table 2:** Defence and immune-related annotated ESTs from turbot libraries

Genes	No. unique sequences	%
Complement related	16	7.9
Apoptosis related	10	4.9
Immunoglobulin related	8	3.9
Glutathione S-transferase	7	3.5
Elastase	6	2.9
Cytochrome P450	6	2.9
Major histocompatibility complex	5	2.5
Coagulation factor	5	2.5
Interferon related	3	1.5
Perforin	3	1.5
Hepcidin precursor	3	1.5
Nephrosin	3	1.5
Alpha-2-macroglobulin	3	1.5
Other genes	119	58.6

Total	203	24.9

"No. unique sequences" refers to the total amount of the different annotated contigs and singletons for each gene class listed in the Table. "Total percentage" of defence/immune-related genes is referred to the number of unique annotated sequences from turbot libraries (816).

Like in mammals, the immune system of fish is composed of non-specific and specific defence. The innate immune response is an important and highly developed defence mechanism against pathogens in fish [43]. Examples of innate immunity include anatomic barriers, mechanical removal of pathogens, bacterial antagonism, pattern-recognition receptors, antigen-nonspecific defence compounds, complement pathways, phagocytosis, and inflammation [43]. In Table 2, a list of defence/immune-related genes that were found three or more times in our libraries is shown. Complement related genes were predominant (7.9%) followed by apoptosis (4.9%) and immunoglobulin (Ig)-related (3.9%) genes. Glutathione S-transferase, heat shock proteins and cytochrome P450, elastases, major histocompatibility complex (MHC) and coagulation factors involved in innate immunity were also present, as well as others like interferon, perforin, hepcidin, nephrosin or alpha-2-macroglobulin. Interestingly, the majority of our immune-related cDNAs (75.0%) were reported for the first time in turbot, even though some of them have important roles in the immune response like B-cell linker, chemotaxin, complement components, IgD, IgM, interferon stimulated gene 12 (b2), lipopolysaccharide (LPS)-binding protein, natural killer (NK)-lysine type 1, peptidoglycan recognition protein, skin mucus lectin and tumour necrosis factor (TNF) receptor associated factor2, among others. Previously, only a hepcidin [44] and a natural killer cell enhancing factor [45] had been characterized in turbot.

The availability of a large number of sequences from immune-related cDNA libraries, both from non-infected fish (controls) as well as from fish challenged with specific pathogens, suggested a comparison of transcript profiles to identify genes regulated in response to these pathogens. Four or more sequences per gene (contig) are the minimum necessary to get statistical power to check the null hypothesis of even distribution of sequences among the three libraries. Ninety six genes presented four or more sequences in our libraries. However, taking into account the large number of tests performed, we decided to use a more conservative set of genes (with 6 or more sequences) to avoid type I errors. This new set comprised 72 genes, whose distribution among libraries is shown in Additional file 1 together with their probability of departure from the null hypothesis using a chi-square test. To use all information available and to get higher statistical power, we decided to perform an additional analysis by comparing the profiles of all genes grouped according to GO categories (see Additional file 2). Though this approach could be mixing genes with opposite expression patterns, it could provide significant trends associated to specific functions for complementing the "individual-level gene" analysis. Bonferroni correction for multiple tests was considered in this case, because of the higher statistical power achieved with this approach.

As expected, defence/immune-related genes appeared overrepresented in the set of 72 genes (37.5%) with regard to the total number of annotated genes in our libraries (24.9%). Some genes apparently responded in a similar way to both pathogens, being down- (myosin, nephrosin and several peptidases) or up-regulated (actin and lysozyme) regarding control. However, most genes responded to only one pathogen. The different infection profile of the pathogens used in this study, a bacterium (*A. salmonicida*) and a parasite (*P. dicentrarchi*), is expected to stimulate a specific set of genes in the host. So, protein biosynthesis was up-regulated in response to *Aeromonas salmonicida*, as suggested the increased amount or ribosome-related and elongation factor proteins, and their correspondent GO terms (see Additional file 2). Also, a general down-regulation of genes involved in proteolytic activity in response to this bacterium (chymotrypsin B precursor, trypsinogen1, chymotrypsinogen 2, trypsynogen-like serin protease and elastase precursor) was observed, in turn correlated to the lower presence of peptidase and proteolysis GO terms in this library. The possibility that *A. salmonicida *could be blocking these genes cannot be ruled out, since it would facilitate the infection process. Additionally, it was noteworthy that several iron metabolism-related genes turned out to be up-regulated after infection with *A. salmonicida *(haptoglobin fragment 1, globin-related proteins, hepcidin precursor). The high relevance of iron for bacteria infection could explain this observation. The up-regulation of hepcidin after bacteria infection has been recently described in turbot and gilthead seabream [44,46]. Finally, some other immune relevant genes like MHC II alpha and beta antigens, complement C9, thrombin, chemotaxin, bactericidal permeability-increasing protein, apolipoprotein A-IV3, heat shock protein 90 beta and chemotaxin showed significant up-regulation in response to *A. salmonicida*.

However, the strongest genetic signature was observed in response to *P. dicentrarchi *infection. Specific genes were exclusively detected in this library and sometimes at high frequencies, like those homologous to *Oryzias latipes *and *Paralichthys olivaceous *cDNA clones and to mitochondrial ATP synthase alpha-subunit gene. Functional annotation of these unknown genes now appears relevant to understand the response of turbot to this pathogen. The same was reflected when GO categories were analyzed (see Additional file 2). A large number of differentially regulated gene categories (*P *= 0) appeared associated to *P. dicentrarchi *library according to the different GO criteria. Cytoskeleton organization and biogenesis, as well as carbohydrate metabolic process appeared up-regulated categories among biological processes. Transporter and hydrolase activities, as well as protein, ion and heme binding also were overexpressed regarding molecular function classification. Finally, cytoskeleton and cytosol appeared as the highest active cellular components. Looking at specific categories, a significant increase in the expression of antioxidant genes, like glutathione peroxidase and glutathione-S-transferase, was observed. These genes play a pivotal role to prevent cellular damage due to the increased reactive oxygen species (ROS) during infection [47]. Paramá et al. [48] have recently demonstrated the increase in intracellular ROS by proteases of turbot kidney in response to this pathogen. Tissue trauma or invasion by pathogens induces changes in the quantities of several macromolecules in animal body fluids, which comprise one aspect of the acute phase response (APR). In fish, APR proteins include pentraxins, serum amyloid P, several components of the complement system, transferrin and thrombin. Up-regulation of pentraxin and serotransferrin was observed in response to *P. dicentrarchi *in our study. Transferrin up-regulation could be related to the increase in inflammation and enhanced oxidative stress typical of infections with this parasite [48]. Finally, up-regulation of other important components of the immune response such the profilin and lysozyme was observed. Profilin-like protein has been reported as a toll-like receptor (TLR) 11 ligand in some parasites [49]. TLRs are evolutionary conserved transmembrane proteins that recognize a unique pattern of molecules derived from pathogens or damaged cells, triggering robust but defined innate immune responses [49].

### Markers-containing ESTs

EST studies can also provide resources for the identification of polymorphic DNA markers such as microsatellites and SNPs. In our study, screening of EST sequences for short tandem repeats (2–6 bp) identified 191 microsatellites using a conservative criterion (≥ 6 and ≥ 8 repeats for tri/tetra/penta and dinucleotide motifs, respectively). Of these, 120 had significant hits in BLAST with e-value cut off ≤ 1e-5 and 71 were annotated. Most microsatellites were dinucleotide (128) and trinucleotide (56), while only 6 tetranucleotide and 1 pentanucleotide were found. Among these 191 ESTs sequences, 104 contained sufficient flanking sequences length for primer design. Fifty of these 104 microsatellites-containing ESTs were contigs, therefore the *in silico *comparison of the sequences included in these contigs allowed us to identify 11 putative polymorphic microsatellites.

Because of their abundance within the genome, SNPs are the most common type of genetic markers [50] for studying complex genetic traits and genome evolution [51]. In turbot there have no been reports on SNP identification so far. The use of non-normalized libraries and a large number of individuals in library construction made possible the identification of 2197 good quality SNPs. After the three filters used in the QualitySNP pipeline [52], we finally detected 2197 real and 1158 true SNPs (Table 3), representing a rate of 1.39 and 0.74 SNPs per 100 bp, respectively. Real and true SNPs included 749 and 453 transitions, 974 and 558 transversions and 366 and 125 indels, respectively. With this pipeline, only clusters with at least 4 EST sequences were selected to minimize the detection of SNPs caused by sequencing errors. As shown in Table 4 the majority of SNPs were detected in contigs involving a larger number of sequences, which provides an additional proof of our SNP quality. The identification of microsatellite and SNPs markers within turbot ESTs will contribute to extend the turbot genetic map [10] after linkage analysis in reference families. Since these markers are linked to genes, they will be useful as Type I markers for population genomics scanning in this species and for comparative mapping and fish evolutionary studies.

**Table 3:** Summary statistics of SNP identification from turbot EST resources

	Real SNPs	True SNPs andSNPs
Total sequences analysed	12584	
Number of contigs	257	255
Total SNPs detected	2197	1158
SNP frequency	1.39/100 bp	0.74/100 bp
Total number of transitions	749	453
C/T	556	344
A/G	193	109
Total number of transversions	974	558
A/T	161	87
A/C	352	214
T/G	251	130
C/G	210	127
Total number of indels	366	125
Tri-allelic polymorphisms	99	21
Tetra-allelic polymorphisms	9	1

Real SNPs are those which passed quality filters 1 and 2 using the pipeline QualitySNP and true SNPs are the highest quality SNPs passing the three filters (see Methods).

**Table 4:** Real and true quality SNP distribution in contigs with 4 or more ESTs

	Number of contigs	Real SNPs	True SNPs
with 4 sequences	22	58	58
with 5–10 sequences	94	295	235
with 11–20 sequences	75	437	266
with 21–30 sequences	28	322	215
with 31–50 sequences	21	289	110
with > 50 sequences	17	796	274

Total	257	2197	1158

Real SNPs are those which passed quality filters 1 and 2 using the pipeline QualitySNP and true SNPs are the highest quality SNPs passing the three filters (see Methods).

## Conclusion

To our knowledge, this is the first report on a large transcriptional analysis in turbot providing new genomic resources in this important European aquaculture species. This study describes a collection of 9256 ESTs representing 3482 unique sequences obtained from three directionally cloned cDNA libraries from *S. maximus*, all of these being novel ESTs for this species. Therefore, this is a valuable EST collection, which increases genomic resources of turbot and enhances the genomic tools available for non-model fish species. The transcript profile comparison among the three libraries allowed the identification of putative genes generally or specifically related with infections in turbot. In addition, a high number of putative microsatellite and SNP EST-markers are now available for turbot map and highly useful for comparative mapping. These ESTs will be the basis for the development of a turbot microarray, focused on the characterization of the transcriptional response to pathogen exposure.

## Methods

### Tissue source and challenge

Two batches of 40 individuals (20–30 g each) obtained from a mixture of heterogeneous genetic families were collected at a specialized turbot fish farm. Fish from each batch were challenged intraperitoneally with *A. salmonicida *subsp. *salmonicida *and *P. dicentrarchi*, respectively [53,54]. The dose was adjusted to obtain around 50% survival (LD50). The challenges were performed at the CETGA facilities in quarantine tanks. Fish were sacrificed prior to organ extraction using a lethal dose of MS222 anesthetic. In order to obtain mRNA representative of both innate and adaptive immune systems across the infection process, liver, spleen and head kidney tissues were collected from 5 sacrificed fish at five sampling points along the infective process for *A. salmonicida *(12 h, 1 day, 3 days, 7 days and 21 days post-inoculation) and at four sampling points for *P. dicentrarchi *(1 day, 3 days, 7 days and 15 days post-inoculation). Analogous batches of control fish were injected with saline serum and sampled at the same days of challenged fish. For each sample time, equal amounts of tissue from liver, kidney and spleen of each fish were pooled and immediately frozen in liquid nitrogen, constituting 15 pools for *A. salmonicida *(3 tissues × 5 sampling points), 12 for *P. dicentrarchi *(3 tissues × 4 sampling points) and the same pools for their respective controls. Each sample of pooled tissues was ground to a fine powder in a mortar and pestled with liquid nitrogen and stored at -80°C until being used for RNA extraction. The use of pools of individuals at each sampling point was planned, both for identifying putative SNPs, as well as for averaging individual effects in future microarray analysis on turbot immune response.

### RNA isolation and cDNA library construction

Total RNA was extracted from pooled tissues of control and infected fish using TRIZOL Reagent (Life Technologies) according to manufacturer's recommendations. RNA quality was assessed in a Bioanalyzer (Bonsai Technologies). RNA was quantified using gel and NanoDrop^® ^ND-1000 spectrophotometer (NanoDrop^® ^Technologies Inc) estimations. Poly-A mRNA was isolated using the Dynabeads^® ^mRNA Purification Kit (INVITROGEN). Three cDNA libraries (from *A. salmonicida *and *P. dicentrarchi *infected fish and control) were directionally constructed (5' *EcoR*I, 3' *Xho*I), with equal amounts of RNA from each tissue at each sampling time, using the ZAP-cDNA Library Construction Kit (STRATAGENE) following manufacturer's instructions except for size fractioning. This was performed on cDNAs prior to ligation into vector and carried out with the SizeSep 400 Spun Columns (GE HEALTHCARE). To allow characterization of the inserts in a plasmid system the library was mass excised according to the manufacturer's recommendations, converting it from plaque forming units to a phagemid in *E. coli *SOLR strain. Manually isolated colonies were randomly picked and arrayed into 96-well microtiter plates containing liquid selective medium. Glycerol stocks of overnight cultures were prepared in 96 well plates and stored at -80°C.

### Sequencing and bioinformatics

Plasmid DNA was isolated from around 4000 clones from each library using the DirectPrep^® ^96 Miniprep kit (QIAGEN) and the Plasmid Miniprep96 kit (MILLIPORE) with a robotic platform (BIOMEK 3000), and checked by electrophoresis in 1% agarose gels. One part of the purified DNA was sequenced following the ABI Prism BigDye™ Terminator v3.1 Cycle Sequencing Kit protocol on an ABI 3100 DNA sequencer (Applied Biosystems) and the other with the DTCS kit protocol on a CEQ2000 DNA sequencer (BECKMAN COULTER). All clones were sequenced from their 3' ends using a standard T7 primer to obtain the highest specific sequences of genes for oligo-microarray design. Those clones which suffered a systematic drop on sequencing signal after poly-A tails were sequenced from 5' end.

A bioinformatic tool was developed in order to process all data. Basecalling from chromatogram traces was performed by using PHRED [55,56]. Vector, poly-A tails and low-quality regions were trimmed from EST sequences using a custom Perl script, a local BLAST search engine and the trimmest utility from the EMBOSS suite. High quality ESTs (at least 100 bp and PHRED score ≥ 20 after removal of vector sequence, adapter, and poly-A tail) of both normal and infected libraries were combined and assembled to form clusters using CAP3 [57] with the overlapping identity percentage and minimum overlapping length parameters set to > 85.0% and 50 bp, respectively, in order to obtain highly reliable contig sequences. These contigs were manually revised to detect possible errors all along the bioinformatics process. ESTs that did not form contigs (singletons) and the contigs resultant of assemblage of multiple sequences were referred to as unique sequences. Singletons and consensus sequences of each contig (unique sequences) were compared against public databases. Unique sequences were searched by both Blastn and Blastx, and the corresponding outputs subsequently parsed using BioPerl for fish-relevant hits and significant UniGene information. GO, KEGG and COG terms were extracted from AutoFACT output, which in turn feeds from Tblastx and RPS-blast output. All results were stored in a mySQL database to be consulted and searched using a custom-designed, friendly AJAX web interface. The e-value cut off was ≤ 1e-5. All annotations thus obtained were complemented with AutoFACT output, whenever available.

### Bioinformatic mining of microsatellites and SNPs

The set of 3482 unique sequences was searched for microsatellites using the program SPUTNIK . The minimum repeat number used for this search was 8 for dinucleotide and 6 for tri-, tetra-and pentanucleotide microsatellites. Microsatellite-containing ESTs were identified as candidates for marker development if they presented enough flanking sequences on either side of the repeats for primer design. Detection of single nucleotide polymorphisms (SNPs) was done using the pipeline QualitySNP with default settings [52]. This program uses three filters for the identification of reliable SNPs: filter 1 screens for all potential SNPs; filter 2 uses a haplotype-based strategy to detect reliable SNPs; and filter 3 screens SNPs by calculating a confidence score based on sequence redundancy and quality. SNPs that pass filters 1 and 2 are called real SNPs, and those that pass all of the three filters are called true SNPs.

## Authors' contributions

This work represents a collaboration between different institutions (Xunta de Galicia -local Government- and Universities) and turbot industry (CETGA- Centro Tecnológico Gallego de Acuicultura-). PM and CB identified the need for this investigation, designed and supervised the project. BGP and AV constructed the cDNA libraries. BGP, CF, and MVE performed all sequencing work, and revised and compiled this information for the subsequent bioinformatics analysis. Database for editing, clustering and functional annotation of genes was developed by JAA, MC, and AG in close connection with PM, BGP, CB, CF and AM. These last two authors performed all bioinformatics analysis using this database. BM and MLL, for *Aeromonas salmonicida*, and JL, for *Philasterides dicentrarchi*, supplied virulent strains and performed all challenges at CETGA facilities. Maintenance, sacrifice, organ and RNA extraction from fish was done by MV and SC. The paper was written by BGP and mostly revised by PM and JAA. All authors read the manuscript and gave their approval.

## Supplementary Material

Additional file 1 **Comparison of gene expression profiles regulated in response to turbot pathogens.** The total number and distribution among libraries (Aeromonas, Philasterides, control) of sequences from contigs with 6 or more sequences is presented. The homology with public databases (e-value), the function and the probability of departure from the null hypothesis of even distribution of sequences among libraries is shown for each contig. Click here for file

Additional file 2 **Comparison of ESTs grouped according to GO terms among the three turbot cDNA libraries.** contigs of annotated genes are shown grouped according to GO term categories (Molecular function; Biological process; Cellular component) and split into the three turbot libraries from which sequences were obtained. The expression pattern of each gene at each library is evaluated as the amount of sequences found. This pattern is compared among libraries and examined for its deviation from an even distribution among libraries by a chi-square test. Click here for file
