# Supplementary material for: Expressed sequence tags (ESTs) from immune tissues of turbot (Scophthalmus maximus) challenged with pathogens
Source: BMC Vet Res. 2008 Sep 25;4:37. doi: 10.1186/1746-6148-4-37 (PMC2569028; doi:10.1186/1746-6148-4-37)
Supplement: Additional file 1 — Comparison of gene expression profiles regulated in response to turbot pathogens. The total number and distribution among libraries (Aeromonas, Philasterides, control) of sequences from contigs with 6 or more sequences is presented. The homology with public databases (e-value), the function and the probability of departure from the null hypothesis of even distribution of sequences among libraries is shown for each contig. [file 1746-6148-4-37-S1.doc]

**Additional file 1 - Comparison of gene expression profiles regulated in response to turbot pathogens**

| **Gene/ GenBank Acc. No.** | **e-value** | **No. Seq.** | **Aerom.** | **Philast.** | **Control** | **Function** | ***P*** |
| --- | --- | --- | --- | --- | --- | --- | --- |
| MHC class II beta antigen mRNA | 0.0 | 9 | 6 | 0 | 3 | Defence/Immune-related | 0.050 |
| Ribosomal protein S6 | 3e-42 | 12 | 6 | 0 | 6 | Other | 0.050 |
| Similar to neurotoxin/C59/Ly-6-like protein *Danio rerio* | 2.e-10 | 21 | 12 | 6 | 3 | Other | 0.050 |
| Carboxipeptidase B | 2.e-15 | 39 | 6 | 18 | 15 | Defence/Immune-related | 0.050 |
| Myosin light chain (Ca binding) | 2.e-84 | 10 | 2 | 1 | 7 | Other | 0.045 |
| Fructose-1,6-bisphosphatase 1 | 4.e-40 | 8 | 1 | 1 | 6 | Other | 0.044 |
| Ribosomal protein S4 | 6.e-54 | 18 | 11 | 4 | 3 | Other | 0.042 |
| Beta actin | 0.0 | 62 | 22 | 28 | 12 | Other | 0.042 |
| Perforin | 5.e-17 | 27 | 3 | 14 | 10 | Defence/Immune-related | 0.032 |
| Ras-related nuclear protein | 1.e-21 | 6 | 5 | 0 | 1 | Defence/Immune-related | 0.030 |
| Glutathione-S-transferase | 5.e-45 | 6 | 0 | 5 | 1 | Defence/Immune-related | 0.030 |
| FE949359 |  | 6 | 5 | 1 | 0 | Unknown | 0.030 |
| Protein disulfide isomerase A3 precursor | 7.e-22 | 6 | 0 | 5 | 1 | Other | 0.030 |
| cDNA clone *Platichthys flesus* | 2.e-13 | 6 | 1 | 5 | 0 | Unknown | 0.030 |
| Nephrosin | 1.e-66 | 6 | 1 | 0 | 5 | Defence/Immune-related | 0.030 |
| Hypothetical protein LOC100136074 *Oncorhynchus mykiss* | 1.e-06 | 8 | 0 | 2 | 6 | Unknown | 0.030 |
| Ribosomal protein S25 | 4.e-26 | 14 | 9 | 4 | 1 | Other | 0.030 |
| Carboxypeptidase A1 | 5.e-63 | 18 | 2 | 5 | 11 | Defence/Immune-related | 0.030 |
| 40S ribosomal protein S30 | 0.0 | 26 | 14 | 3 | 9 | Other | 0.030 |
| Lysozime C precursor | 8.e-42 | 76 | 22 | 36 | 18 | Defence/Immune-related | 0.029 |
| Antifreeze protein type IV | 3.e-30 | 22 | 13 | 6 | 3 | Defence/Immune-related | 0.028 |
| FE951739 |  | 10 | 0 | 7 | 3 | Unknown | 0.025 |
| Sulfated glycoprotein | 2.e-09 | 10 | 0 | 3 | 7 | Other | 0.025 |
| Ribosomal protein L35a | 6.e-51 | 10 | 3 | 0 | 7 | Other | 0.025 |
| Beta-microseminoprotein A1 precursor | 2.e-31 | 14 | 0 | 8 | 6 | Other | 0.024 |
| Hemoglobin beta-2 subunit | 2.e-38 | 13 | 5 | 0 | 8 | Other | 0.023 |
| Chymotrypsinogen 2 | 4.e-46 | 50 | 8 | 18 | 24 | Other | 0.020 |
| Serotransferrin | 5.e-11 | 88 | 18 | 40 | 30 | Defence/Immune-related | 0.016 |
| Ribosomal protein S2 | 0.0 | 171 | 75 | 49 | 47 | Other | 0.014 |
| Glutathion peroxidase 1 | 4.e-28 | 9 | 0 | 7 | 2 | Defence/Immune-related | 0.013 |
| MHC II alpha antigen | 6.e-36 | 7 | 6 | 0 | 1 | Defence/Immune-related | 0.012 |
| Ribosomal protein L28 | 0.0 | 7 | 6 | 0 | 1 | Other | 0.012 |
| FE951925 |  | 7 | 1 | 6 | 0 | Unknown | 0.012 |
| Fatty acid-binding protein | 3.e-56 | 7 | 0 | 1 | 6 | Other | 0.012 |
| Ribosomal protein S27a precursor | 1.e-53 | 16 | 11 | 3 | 2 | Other | 0.010 |
| Elastasa 2 precursor | 1.e-64 | 24 | 5 | 4 | 15 | Defence/Immune-related | 0.010 |
| Trypsinogen-like serine protease | 3.e-26 | 60 | 9 | 27 | 24 | Defence/Immune-related | 0.010 |
| NADH dehydrogenase subunit 4 | 8.e-110 | 13 | 4 | 0 | 9 | Other | 0.009 |
| Ribosomal protein L13a | 5.e-73 | 13 | 9 | 0 | 4 | Other | 0.009 |
| Haptoglobin fragment 1 | 6.e-12 | 38 | 19 | 15 | 4 | Defence/Immune-related | 0.009 |
| cDNA clone Hippoglossus hippoglossus | 1.e-56 | 5 | 5 | 0 | 0 | Unknown | 0.007 |
| Bactericidal permeability-increasing protein/lipopolysaccharide (LPS)-binding protein | 1.e-07 | 5 | 5 | 0 | 0 | Defence/Immune-related | 0.007 |
| Ethylmalonic encephalopathy 1 | 1.e-32 | 5 | 0 | 5 | 0 | Defence/Immune-related | 0.007 |
| cDNA clone *Dicentrarchus labrax* | 5.e-35 | 5 | 0 | 5 | 0 | Unknown | 0.007 |
| FE951614 |  | 5 | 0 | 5 | 0 | Unknown | 0.007 |
| Transcription factor BTF3 | 2.e-43 | 5 | 0 | 0 | 5 | Other | 0.007 |
| Apolipoprotein A-IV3 | 1.e-36 | 5 | 5 | 0 | 0 | Defence/Immune-related | 0.007 |
| cDNA clone *Gasterosteus aculeatus* | 2.e-77 | 5 | 0 | 0 | 5 | Unknown | 0.007 |
| FE951458 |  | 8 | 0 | 7 | 1 | Unknown | 0.005 |
| Chemotaxin | 3.e-33 | 13 | 10 | 2 | 1 | Defence/Immune-related | 0.004 |
| Elongation factor 1-alpha | 2.e-25 | 36 | 21 | 5 | 10 | Other | 0.004 |
| FE948311 |  | 9 | 0 | 8 | 1 | Other | 0.002 |
| Complement C9 | 8.e-50 | 11 | 9 | 0 | 2 | Defence/Immune-related | 0.002 |
| Heat shock protein 90 beta | 2.e-53 | 25 | 16 | 2 | 7 | Defence/Immune-related | 0.002 |
| TBT-binding protein | 2.e-10 | 32 | 20 | 6 | 6 | Defence/Immune-related | 0.002 |
| cDNA clone *Paralichthys olivaceus* | 1.e-23 | 7 | 0 | 7 | 0 | Unknown | 0.001 |
| Mitochondrial ATP synthase alpha-subunit | 5.e-57 | 7 | 0 | 7 | 0 | Other | 0.001 |
| Thrombin B chain | 3.e-101 | 10 | 9 | 0 | 1 | Defence/Immune-related | 0.001 |
| Hemoglobin alpha-1 chain | 6.e-32 | 219 | 100 | 57 | 62 | Other | 0.001 |
| FE951239 |  | 9 | 0 | 9 | 0 | Unknown | 0.000 |
| Pentraxin | 8.e-65 | 13 | 1 | 11 | 1 | Defence/Immune-related | 0.000 |
| cDNA clone *Oryzias latipes* | 8.e-34 | 17 | 0 | 17 | 0 | Unknown | 0.000 |
| Elastase 3 precursor | 4.e-78 | 20 | 0 | 16 | 4 | Other | 0.000 |
| Chymotrypsin B precursor | 2.e-83 | 37 | 3 | 8 | 26 | Other | 0.000 |
| Profilin 2 | 3.e-12 | 39 | 1 | 32 | 6 | Defence/Immune-related | 0.000 |
| Trypsinogen 1 | 1.e-90 | 47 | 5 | 15 | 27 | Other | 0.000 |
| Antifreeze polypeptide (AFP) precursor | 3.e-06 | 52 | 7 | 15 | 30 | Defence/Immune-related | 0.000 |
| Hepcidin precursor | 3.e-21 | 70 | 50 | 13 | 7 | Defence/Immune-related | 0.000 |
| Trypsinogen 1 precursor | 7.e-21 | 86 | 17 | 47 | 22 | Other | 0.000 |
| Trypsinogen 1 | 4.e-88 | 104 | 18 | 35 | 51 | Other | 0.000 |
| 14 kDa apolipoprotein | 1e -5 | 148 | 33 | 82 | 33 | Other | 0.000 |
| Beta type globin | 3e-127 | 407 | 70 | 212 | 125 | Other | 0.000 |

The total number of sequences identified at each contig (No. seq.) is split into the three libraries: *A. salmonicida* (Aerom.), *P. dicentrarchi* (Philast.) and control. Genes were classified as defence/immune-related when such relationship was previously reported in vertebrate scientific literature. *P* values, ordered from top to bottom, refer to deviation from the null hypothesis of even distribution of sequences among the three libraries using a chi-square test. The GenBank accession number of the largest sequence of the contig is included at those genes without matching in public databases.
