# Supplementary material for: Expressed sequence tags (ESTs) from immune tissues of turbot (Scophthalmus maximus) challenged with pathogens
Source: BMC Vet Res. 2008 Sep 25;4:37. doi: 10.1186/1746-6148-4-37 (PMC2569028; doi:10.1186/1746-6148-4-37)
Supplement: Additional file 2 — Comparison of ESTs grouped according to GO terms among the three turbot cDNA libraries. contigs of annotated genes are shown grouped according to GO term categories (Molecular function; Biological process; Cellular component) and split into the three turbot libraries from which sequences were obtained. The expression pattern of each gene at each library is evaluated as the amount of sequences found. This pattern is compared among libraries and examined for its deviation from an even distribution among libraries by a chi-square test. [file 1746-6148-4-37-S2.doc]

**Additional file 2 - Comparison of ESTs grouped according to GO terms among the three turbot libraries**

| **A. Biological proccess** |  |  |  |  |  |  |  |  |
| --- | --- | --- | --- | --- | --- | --- | --- | --- |
| **GO terms** | **No Ids** | **Contigs** | **Singletons** | **Sequences** | **Aerom.** | **Philast.** | **Control** | ***P*** |
| Biosyntethic process | 100 | 82 | 18 | 1270 | 503 | 349 | 418 | **0,000** |
| Metabolic process | 33 | 19 | 14 | 210 | 74 | 81 | 55 | 0,075 |
| Protein metabolic process | 62 | 37 | 25 | 755 | 314 | 222 | 219 | **0,000** |
| Regulation of biological process | 8 | 3 | 5 | 20 | 3 | 6 | 11 | 0,086 |
| Cellular process | 8 | 6 | 2 | 112 | 36 | 45 | 31 | 0,260 |
| Immune system process | 16 | 10 | 6 | 58 | 29 | 15 | 14 | 0,026 |
| Primary metabolic process | 24 | 11 | 13 | 112 | 40 | 41 | 31 | 0,444 |
| Lipid metabolic process | 10 | 5 | 5 | 16 | 6 | 8 | 2 | 0,174 |
| Cellular metabolic process | 8 | 5 | 3 | 40 | 13 | 9 | 18 | 0,218 |
| Developmental process | 8 | 1 | 7 | 9 | 2 | 1 | 6 | 0,097 |
| Cellular homeostasis | 11 | 6 | 5 | 148 | 42 | 56 | 50 | 0,368 |
| Transport | 25 | 17 | 8 | 438 | 179 | 123 | 136 | 0,003 |
| Ion trasnport | 13 | 10 | 3 | 142 | 33 | 65 | 44 | 0,004 |
| Electron trasnport | 32 | 20 | 12 | 294 | 111 | 89 | 94 | 0,257 |
| Protein transport | 9 | 6 | 3 | 36 | 18 | 4 | 14 | 0,013 |
| Lipid transport | 5 | 3 | 2 | 248 | 119 | 66 | 63 | **0,000** |
| Proteolysis | 72 | 47 | 25 | 803 | 165 | 275 | 363 | **0,000** |
| Nucleobase, nucleoside, nucleotide and nucleic acid metabolic process | 44 | 22 | 22 | 120 | 41 | 39 | 40 | 0,975 |
| Response to stress | 19 | 13 | 6 | 198 | 86 | 67 | 45 | 0,002 |
| Response to stimulus | 4 | 1 | 3 | 5 | 2 | 2 | 1 | 0,819 |
| Signal transduction | 22 | 13 | 9 | 145 | 59 | 47 | 39 | 0,123 |
| Cytoskeleton organization & biogenesis | 9 | 6 | 3 | 73 | 12 | 46 | 15 | **0,000** |
| Organelle organization & biogenesis | 8 | 4 | 4 | 44 | 19 | 7 | 18 | 0,049 |
| Chromosome organization & biogenesis | 6 | 1 | 5 | 7 | 4 | 3 | 0 | 0,156 |
| Transcription | 31 | 9 | 22 | 70 | 27 | 17 | 26 | 0,273 |
| Cell differentiation | 11 | 4 | 7 | 118 | 48 | 45 | 25 | 0,019 |
| Reproduction | 7 | 3 | 4 | 14 | 5 | 3 | 6 | 0,607 |
| Cell adhesion | 7 | 4 | 3 | 18 | 7 | 6 | 5 | 0,846 |
| Pathogenesis | 5 | 2 | 3 | 9 | 0 | 5 | 4 | 0,097 |
| Biological adhesion | 5 | 2 | 3 | 9 | 0 | 5 | 4 | 0,097 |
| Carbohydrate metabolic process | 14 | 7 | 7 | 436 | 76 | 224 | 136 | **0,000** |
| Biological process | 6 | 0 | 6 | 6 | 3 | 1 | 2 | 0,607 |
| **B. Molecular Function** |  |  |  |  |  |  |  |  |
| **GO terms** | **No Ids** | **Contigs** | **Singletons** | **Sequences** | **Aeromonas** | **Philasterides** | **Control** | ***P*** |
| Structural constituent of ribosome | 96 | 79 | 17 | 1249 | 488 | 344 | 417 | **0,000** |
| Transferase activity | 35 | 18 | 17 | 85 | 29 | 32 | 24 | 0,562 |
| Motor activity | 5 | 2 | 3 | 15 | 3 | 4 | 8 | 0,247 |
| Translation factor activity, nucleic acid binding | 10 | 8 | 2 | 87 | 40 | 20 | 27 | 0,029 |
| Enzyme regulator activity | 21 | 18 | 3 | 202 | 61 | 73 | 68 | 0,583 |
| Oxidoreductase activity | 74 | 43 | 31 | 465 | 178 | 144 | 143 | 0,077 |
| Transporter activity | 33 | 24 | 9 | 807 | 230 | 330 | 247 | **0,000** |
| Structural molecule activity | 24 | 9 | 15 | 46 | 14 | 15 | 17 | 0,859 |
| Hydrolase activity | 53 | 29 | 24 | 658 | 156 | 308 | 194 | **0,000** |
| Transcription regulator activity | 11 | 4 | 7 | 17 | 6 | 7 | 4 | 0,662 |
| Kinase activity | 13 | 6 | 7 | 24 | 11 | 8 | 5 | 0,325 |
| Catalytic activity | 12 | 6 | 6 | 48 | 12 | 19 | 17 | 0,444 |
| Receptor activity | 10 | 4 | 6 | 33 | 11 | 12 | 10 | 0,913 |
| Isomerase activity | 7 | 1 | 6 | 8 | 4 | 1 | 3 | 0,417 |
| Lyase activity | 8 | 4 | 4 | 35 | 13 | 10 | 12 | 0,819 |
| Chemokine activity | 6 | 3 | 3 | 15 | 5 | 5 | 5 | 1,000 |
| RNA binding | 37 | 25 | 12 | 411 | 170 | 109 | 132 | **0,001** |
| Protein binding | 88 | 49 | 39 | 920 | 250 | 379 | 291 | **0,000** |
| Calcium ion binding | 22 | 11 | 11 | 87 | 38 | 24 | 25 | 0,122 |
| Peptidase activity | 71 | 47 | 24 | 802 | 166 | 274 | 362 | **0,000** |
| Nucleic acid binding | 12 | 2 | 10 | 16 | 8 | 2 | 6 | 0,174 |
| Cofactor binding | 10 | 4 | 6 | 41 | 19 | 14 | 8 | 0,109 |
| Ion binding | 80 | 49 | 31 | 1156 | 339 | 453 | 364 | **0,000** |
| Heme binding | 12 | 9 | 3 | 743 | 218 | 301 | 224 | **0,000** |
| Binding | 24 | 13 | 11 | 110 | 38 | 39 | 33 | 0,754 |
| DNA binding | 33 | 10 | 23 | 62 | 18 | 19 | 25 | 0,500 |
| Lipid binding | 12 | 8 | 4 | 272 | 127 | 70 | 75 | **0,000** |
| Nucleotide binding | 54 | 27 | 27 | 199 | 86 | 54 | 59 | 0,011 |
| Carbohydrate binding | 8 | 5 | 3 | 92 | 17 | 31 | 44 | 0,003 |
| Oxigen binding | 7 | 5 | 2 | 255 | 113 | 65 | 77 | **0,001** |
| **C. Cellular component** |  |  |  |  |  |  |  |  |
| **GO terms** | **No Ids** | **Contigs** | **Singletons** | **Sequences** | **Aerom.** | **Philast.** | **Control** | ***P*** |
| Ribosome | 98 | 81 | 17 | 1287 | 499 | 358 | 430 | **0,000** |
| Cytoskeleton | 28 | 18 | 10 | 556 | 97 | 288 | 171 | **0,000** |
| Nucleus | 59 | 19 | 40 | 122 | 50 | 38 | 34 | 0,182 |
| Cytoplasm | 48 | 23 | 25 | 219 | 84 | 70 | 65 | 0,265 |
| Mitochondrion | 35 | 24 | 11 | 345 | 127 | 100 | 118 | 0,193 |
| Extracellular region | 44 | 23 | 21 | 580 | 230 | 186 | 164 | **0,003** |
| Cytosol | 21 | 11 | 10 | 703 | 203 | 292 | 208 | **0,000** |
| Membrane | 84 | 48 | 36 | 480 | 178 | 155 | 147 | 0,198 |
| Chromosome | 11 | 4 | 7 | 23 | 10 | 5 | 8 | 0,438 |
| Macromolecular complex | 16 | 11 | 5 | 48 | 17 | 17 | 14 | 0,829 |
| Intracellular | 10 | 5 | 5 | 15 | 3 | 4 | 8 | 0,247 |

No Ids: number of unique sequences; *P*: probability of departure from even distribution of sequences among libraries using a chi-square test.

In bold significant deviations after Bonferroni correction.
